# Quantum chemical benchmark databases of gold-standard dimer interaction energies

**DOI:** 10.1038/s41597-021-00833-x

**Published:** 2021-02-10

**Authors:** Alexander G. Donchev, Andrew G. Taube, Elizabeth Decolvenaere, Cory Hargus, Robert T. McGibbon, Ka-Hei Law, Brent A. Gregersen, Je-Luen Li, Kim Palmo, Karthik Siva, Michael Bergdorf, John L. Klepeis, David E. Shaw

**Affiliations:** 1 D. E. Shaw Research, New York, NY 10036 USA; 2 Department of Biochemistry and Molecular Biophysics, Columbia University, New York, NY 10032 USA

**Keywords:** Scientific data, Quantum chemistry, Computational models, Quantum mechanics, Computational biophysics

## Abstract

Advances in computational chemistry create an ongoing need for larger and higher-quality datasets that characterize noncovalent molecular interactions. We present three benchmark collections of quantum mechanical data, covering approximately 3,700 distinct types of interacting molecule pairs. The first collection, which we refer to as DES370K, contains interaction energies for more than 370,000 dimer geometries. These were computed using the coupled-cluster method with single, double, and perturbative triple excitations [CCSD(T)], which is widely regarded as the gold-standard method in electronic structure theory. Our second benchmark collection, a core representative subset of DES370K called DES15K, is intended for more computationally demanding applications of the data. Finally, DES5M, our third collection, comprises interaction energies for nearly 5,000,000 dimer geometries; these were calculated using SNS-MP2, a machine learning approach that provides results with accuracy comparable to that of our coupled-cluster training data. These datasets may prove useful in the development of density functionals, empirically corrected wavefunction-based approaches, semi-empirical methods, force fields, and models trained using machine learning methods.

## Abstract (summary)

**Table:** 

Measurement(s)	Molecular Interaction Process • interaction energy • energy
Technology Type(s)	ab initio quantum chemistry computational method
Factor Type(s)	molecular entity

Machine-accessible metadata file describing the reported data: 10.6084/m9.figshare.13521638

## Background & Summary

Noncovalent interactions are essential determinants of the properties of molecular liquids and crystals, solvation effects, and the structure and function of biomolecules. Experimental means of quantifying individual noncovalent interactions are limited to small systems with relatively rigid intramolecular degrees of freedom^1^, and computer simulations offer a much-needed alternative; quantum mechanical (QM) calculations, for example, enable the characterization of noncovalent interactions with high accuracy. Among QM-based approaches, the use of coupled-cluster singles and doubles with perturbative triples [CCSD(T)]^2–4^ at the complete basis set (CBS) limit is widely recognized as the gold-standard method for noncovalent interactions^4^.

High-accuracy QM methods come with an intrinsically high cost; CCSD(T), for example, scales as *O(N*^7^) with system size. Publicly available databases^5–14^ offer a way to amortize this cost over a large user community, thus reducing the burden on individual researchers. Such databases serve as recognized benchmarks, and are indispensable resources for both accuracy assessment and parameterization of more affordable QM approximations such as exchange-correlation functionals^12–17^ in the density functional theory framework, empirically corrected wavefunction-based approaches^18–23^, and semi-empirical methods^24–27^ (for a comprehensive review, see summary works^28,29^). Benchmark-quality QM data, often in combination with experimental data, also feature prominently in the development of many empirical molecular mechanics–based models (so-called “force fields”)^30–34^. Diverse, extensive, and consistent collections of high-quality data, moreover, can enable powerful machine learning approaches to be leveraged for molecular modeling^35–41^.

Here we present three benchmark databases of quantum chemical data, including the full Cartesian coordinates of the associated geometries^42^. The first is DES370K, a database of dimer interaction energies computed at the CCSD(T)/CBS level of theory. This database features 370,959 unique geometries for 3,691 distinct dimers, which represent 392 closed-shell chemical species (both neutral molecules and ions) including, but not limited to, water and the functional groups found in proteins. An important subset of the data in the DES370K collection consists of QM-optimized dimer structures, which were used as starting points to generate additional structures along one-dimensional radial profiles. To enhance orientational diversity and ensure adequate sampling of the internal degrees of freedom in the larger chemical species, the dataset also includes a large ensemble of structures (and corresponding radial profiles) obtained from molecular dynamics (MD) simulations (Table 1). Because many potential applications of the presented data, such as parameterizing a new exchange-correlation functional, are computationally demanding, we additionally compiled DES15K, a core subset of the most representative structures from DES370K that largely retains the chemical and orientational diversity of DES370K, but with reduced resolution of scan points in the radial profiles (Table 1).

**Table 1:** Summary information for the DES370K, DES15K, and DES5M databases.

Database	Protocol	Monomers	Dimers	Groups	Dimer geometries
DES370K	Dimer scans based on QM optimization^a^	166	3,436	3,476	97,368
	Dimer scans based on MD configurations^b^	382	466	6,133	166,914
	Homodimer single points based on MD configurations^c^	91	91	910	42,201
	Heterodimer single points based on MD configurations^d^	261	261	2,150	64,476
	Total	392	3,691	12,669	370,959
DES15K (subset of DES370K)	Dimer scans based on QM optimization^a^	159	3,052	3,052	12,183
	Dimer scans based on MD configurations^b^	137	206	1,929	2,468
	Total	159	3,052	4,981	14,651
DES5M	Dimer scans based on QM optimization^a^	153	2,826	71,847	2,404,926
	Dimer scans based on MD configurations^b^	159	328	47,648	1,646,832
	Homodimer single points based on MD configurations^c^	138	138	12,983	464,951
	Heterodimer single points based on MD configurations^d^	163	163	14,641	439,229
	Total	206	2,967	147,119	4,955,938

For each database, we list the protocols employed to generate particular subsets of the data, counts associated with those subsets, and the total count across subsets. The counts shown are the number of chemically distinct monomer types (“Monomers”); the number of chemically distinct dimer types (“Dimers”); the number of groups (“Groups”), where a group is a set of connected calculations, such as those from a radial profile under a dimer-scan protocol or those from a single MD frame under a single-point protocol; and the total number of dimer calculations (i.e., entries in the database) (“Dimer geometries”).

^a^Reference dimer geometries were identified using QM optimization and used to construct a group of radial scan–based geometries.

^b^Reference dimer geometries were extracted from MD simulations of neat liquids and solvated monomers and used to construct a group of radial scan–based geometries.

^c^Reference multimer geometries were extracted from MD simulations of neat liquids and decomposed into a group of single-point dimer geometries.

^d^Reference multimer geometries were extracted from MD simulations of solvated monomers and decomposed into a group of single-point dimer geometries.

The DES370K collection was the source of both training and test data for a machine learning method, SNS-MP2, which we have described in full detail elsewhere^39^. Briefly, the SNS-MP2 approach combines the spin-component-scaled second-order Møller-Plesset perturbation theory (MP2) method^43^ with a neural network to predict per-conformer same-spin and opposite-spin energy scaling coefficients. We found^39^ that for dimer interaction energies, the SNS-MP2 method offers—at a greatly reduced cost—accuracy comparable to that of the CCSD(T)/CBS approach used to obtain the benchmark data in DES370K. The SNS-MP2 neural network also provides per-conformer confidence intervals for the predicted interaction energies^39^.

Using the SNS-MP2 approach, we generated DES5M, a database of predicted gold-standard dimer interaction energies and their associated confidence intervals (Table 1). The DES5M collection contains 4,955,938 additional unique geometries originating from the same two sources as were used for DES370K: radial profiles starting from a set of QM-optimized conformers and dimer geometries extracted from MD simulations. Both the DES5M and DES370K databases also include the full set of MP2-based QM observables that serve as inputs to the SNS-MP2 procedure^39^, thereby allowing for the parameterization and evaluation of other SNS-MP2-like models.

We expect that these three databases will serve as valuable benchmarks for a variety of approximate methods in computational chemistry.

## Methods

### Generation of monomer geometries

Input monomers were specified in the simplified molecular-input line-entry system (SMILES) string format^44^. Hydrogen atoms were added and initial three-dimensional (3D) conformations were generated using the Open Babel^45^ software package. The geometry was then optimized using the OPLS_2005 force field^46^, starting from a large number of perturbed initial structures (with dihedral angles sampled randomly from a uniform distribution over the range ±180° and out-of-plane angles sampled randomly from a uniform distribution over the range ±30°), to identify a set of unique stable conformers for each monomer.

Our intent was to use the QM data to fit force fields for MD simulation, and so we followed the common practice of constraining bonds to hydrogen atoms and valent angles involving two hydrogens to predefined target values. These constraints lead to more stable MD simulations, thus enabling the use of larger time steps. For a bond length, the target value was derived as a sum of three contributions: the equilibrium distance (*R*_*e*_); a vibrational correction, which accounts for the anharmonicity of the stretch potential; and a correction to account for condensed-phase effects in water. The vibrational correction was estimated by approximating the monomer energy with a Morse potential^47^ as a function of the bond length, $U\left(R\right)={D}_{e}{\left(1-{e}^{-\alpha \left(R-{R}_{e}\right)}\right)}^{2}$, where *D*_*e*_ and *α* are fitted parameters that control the well depth and width of the potential, respectively. The equation for the Morse potential leads to the following relationship between the equilibrium (*R*_*e*_) and vibrationally averaged (*R*_*g*_) bond lengths: ${R}_{g}-{R}_{e}=3\hbar /4\sqrt{2{D}_{e}\mu }$, where ћ denotes the Planck constant and *μ* the reduced mass of the two bonded atoms. The condensed-phase effects were estimated from the energy derivative along the stretch coordinate in a system containing the molecule of interest surrounded by a 4-Å-thick shell of solvent molecules (typically consisting of 16–26 waters). Constraint targets for valent angles were derived from their equilibrium values corrected for the condensed phase effect. We omitted angle vibrational corrections, which we expected to have only a small impact on intermolecular interactions.

We subjected our set of force field–derived monomer conformations to QM-geometry optimization, applying constraints to hydrogen-containing bond lengths and angles, at the MP2 level of theory using the density-fitting, local, and frozen-core approximations (DF-LMP2)^48–58^ in the MOLPRO 2012 quantum software package (http://www.molpro.net)^59^ with a triple-zeta, correlation-consistent basis set (aVTZ). (A detailed description of this basis set, and all other basis sets used in this study—including the double-zeta (aVDZ) and quadruple-zeta (aVQZ) variants of aVTZ—is provided in the Supplementary Information.)^60–76^ The resulting set of unique monomer conformations were the starting point for the generation of QM-based dimer geometries.

### Generation of QM-based dimer geometries

Dimer geometries were initially optimized with the OPLS_2005^46^ force field starting from randomly generated relative monomer positions and orientations; the monomer conformations themselves were randomly selected from the corresponding set of QM-optimized structures (described above). The monomers were kept rigid during both this step and all subsequent QM dimer optimization steps. We identified unique dimer minima from this set and then optimized them using a two-step QM procedure: first at the relatively inexpensive DF-LMP2/aVDZ level of theory, then at the DF-MP2/aVTZ level (the convergence threshold for rigid-body optimization was 10^−4^ a.u. in both the center-of-mass gradient and torque). We note that because these minima are seeded from an empirical force field, we do not expect to necessarily recapitulate the global minimum as captured by a higher-level QM method. The set of unique QM-optimized dimer geometries served as starting points for one-dimensional radial scans along an intermolecular axis in 0.1-Å steps, probing separations that were either more compact (i.e., with the shortest intermolecular contact reaching ~1 Å) or more distant (i.e., up to 5 Å more distant than the reference). The internal monomer geometries were preserved when constructing these scans. The intermolecular axis was defined as the line connecting weighted atomic centers of the two molecules, with the weight for each atom defined as *C*/*R*^6^, where *R* is the distance to the nearest atom from the other molecule (coefficient *C* is 1.0 for heavy atoms and 0.1 for hydrogens). Such a definition successfully reproduces, in an automated way, intuitively expected dissociation directions for both nonpolar complexes and hydrogen-bonded dimers; for example, in the latter case the two monomer centers reside in the vicinity of the donor and acceptor atoms.

### Generation of MD-based dimer geometries

To more closely mimic biologically relevant physical conditions, we derived a large set of dimer geometries from condensed-phase MD simulations. For a given molecule, two types of simulations were run (both with the OPLS_2005 force field and MD sampling under the NVT ensemble using the Desmond software package)^77,78^: First, a neat liquid was simulated at the temperature closest to 298 K under which the system remains a liquid at atmospheric pressure, with the density set to the experimentally determined value for that liquid; second, a single solute molecule solvated in a cubic water box (30 Å × 30 Å × 30 Å) was simulated at 298 K and a pre-solute density of 0.997 g cm^−3^. Dimer configurations were extracted from the MD simulation frames and clustered as follows: (i) randomly select an MD dimer configuration as a center of the first cluster and remove from the ensemble *M*/*N* structures closest to the center, where *M* is the number of MD configurations and *N* is the desired number of clusters; (ii) select as a center of the second cluster the configuration most distant from the first center and remove from the remaining unassigned ensemble *M*/*N* structures closest to the second center; (iii) repeat step (ii) until *N* centers are selected based on the largest distance to the closest previously selected center. The distance between two conformers is defined according to the bag-of-bonds^79^ approach. Such a procedure achieves the twin objective of obtaining samples that are both representative and diverse. These dimer configurations were then used to generate radial scans following the same protocol as for QM-optimized conformers.

Multimer configurations were typically extracted from the same MD simulation frames. The multimer configurations extracted from neat liquid simulations were decomposed into the set of all possible homodimer geometries, and those extracted from the water-solvated monomer simulations were decomposed into the set of all possible heterodimer geometries (unless water was both the solute and solvent, in which case water dimer geometries were generated). These multimer-derived dimer configurations were used in single-point QM calculations (not used to seed radial scans).

### QM calculation of dimer interaction energies

For all dimer geometries (including at every point along each radial scan), the interaction energy was computed at the DF-MP2/aVQZ level of theory and counterpoise-corrected for basis-set-superposition error (BSSE)^80^. The resulting MP2 interaction energies form the basis of all datasets presented herein.

The DES370K dataset, which includes CCSD(T) interaction energies, was constructed using the QM- and MD-based protocols for generating dimer geometries described above, but with a more limited set of conformers. QM-derived dimer configurations were restricted to the scans containing the most stable dimer structure for each chemically distinct dimer type. In the case of MD-derived dimer configurations, the number of scans and multimers was limited to ~10 for each chemically distinct dimer type included in the dataset. We excluded the most compact, and thus very repulsive, conformers from all scans.

For each dimer in the DES370K dataset, we calculated a benchmark CCSD(T) interaction energy by using the “gold-standard” method of combining canonical MP2 energies extrapolated to the CBS limit with the difference between the CCSD(T) and MP2 energy estimated in a smaller basis set^5^. For MP2/CBS extrapolation, we used a two-point extrapolation^81^ of DF-MP2/aVTZ and DF-MP2/aVQZ counterpoise-corrected interaction energies. The post-MP2 interaction energy correction (denoted ΔCCSD(T)) was estimated by the difference between counterpoise-corrected CCSD(T) and MP2 interaction energies in the largest basis set that we could afford; this basis set varied from aVQZ for the smallest systems (e.g., a water dimer) to aVDZ for the largest (e.g., a phenol dimer).

**Fig. 1:**
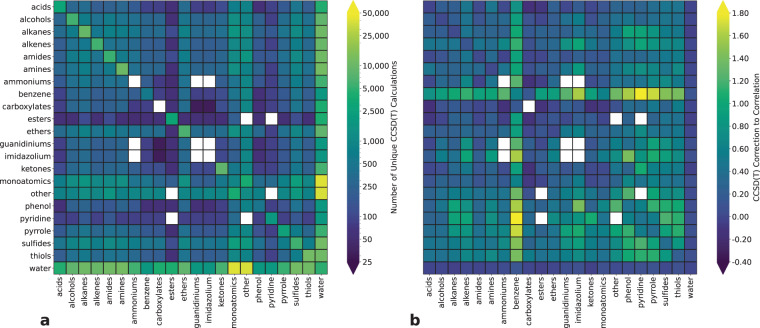
Heatmaps of (**a**) dimer counts and (**b**) ΔCCSD(T) in kcal mol^−1^ for DES370K. The rows and columns of the matrices correspond to the molecule classes of the two monomers. A full list of the monomer SMILES strings assigned to each molecule class is provided in the Supplementary Information. Figure made with Matplotlib^93^ and Seaborn^94^.

Figure 1 shows heatmaps of dimer counts and ΔCCSD(T) for DES370K, grouped according to the molecule class of the two monomers. (A full list of SMILES strings assigned to each molecule class is provided in the Supplementary Information).

### SNS-MP2 predictions

For every dimer included in DES5M, the interaction energy was computed using our SNS-MP2 approach, described in full detail elsewhere^39^. In addition to predicting an energy value, SNS-MP2 quantifies the uncertainty of that prediction: Each SNS-MP2 energy is accompanied by the upper and lower bounds of a 90% confidence interval associated with that prediction.

### Calculation of other QM quantities

Beyond MP2 energies, SNS-MP2 requires additional features that encode the interaction in a geometry-independent manner, leveraging commonalities between chemically disparate dimers. An account of all inputs to the neural network can be found elsewhere^39^; here we describe only these additional quantities, which are included as entries in all three datasets. All of the below quantities are calculated automatically when using the SNS-MP2 plugin^39^ (https://github.com/DEShawResearch/sns-mp2) which relies on the Psi4 quantum chemistry software package^82^.

For each monomer in our set of dimers, we calculated the Hartree-Fock (HF) wavefunction (and thus density matrix) and the MP2 density matrix in the monomer basis. From these quantities, we calculated three properties of the dimer interaction: the classical electrostatic interaction energy, the Heitler-London energy, and the density-matrix overlap.

For each dimer, we calculated the following SAPT0/aVTZ energy components^82–86^: the second-order dispersion, induction, exchange-dispersion, and exchange-induction energies; the same-spin component of the second-order dispersion and exchange-dispersion energies; the first-order electrostatic and exchange energies; and the first-order exchange energy computed in the *S*^2^ approximation.

**Fig. 2:**
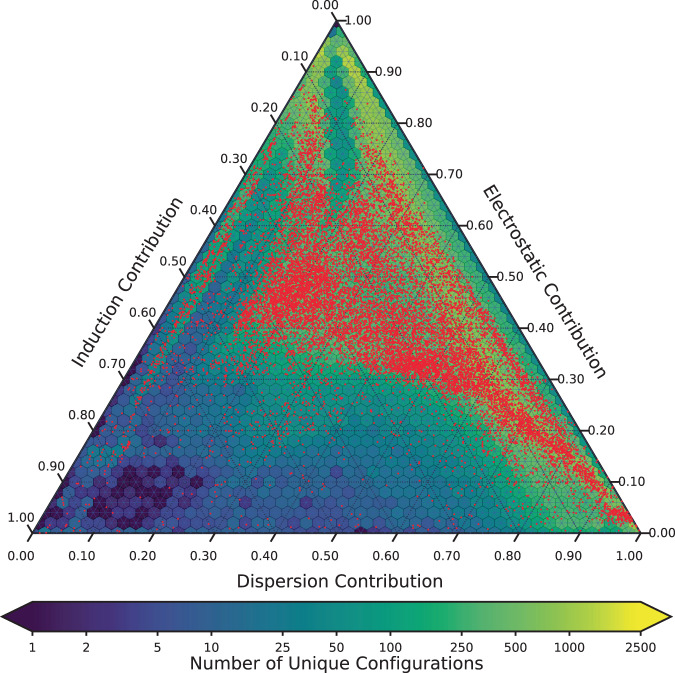
Ternary plot showing the relationship between the electrostatic, dispersion, and induction energy components, as calculated using SAPT, for the dimers in DES370K and DES15K. Counts for DES370K are colored according to the color bar, and DES15K dimers are indicated by red points. Figure made with Matplotlib^93^ and Python-Ternary^95^.

Figure 2 portrays a SAPT interaction energy analysis of the DES370K and DES15K datasets.

### Core subset DES15K

**Fig. 3:**
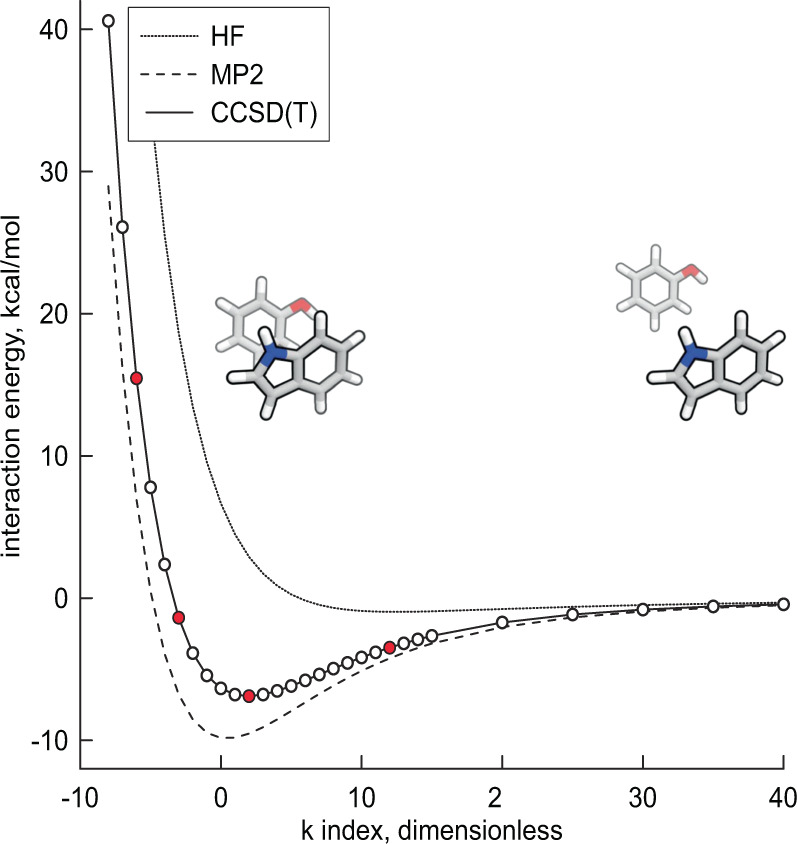
Typical dimer interaction energy profile, showing HF (at aVQZ), MP2 (at the CBS limit), and CCSD(T) (computed using the hybrid “gold-standard^5^” method with ΔCCSD(T)/aVDZ) interaction energies. The plot corresponds to the most stable phenol-indole dimer (obtained using QM optimization), but is representative of other dimer scans. The x-axis shows the k index, with k = 0 corresponding to the reference geometry (in this case, the most stable QM-optimized geometry, based on MP2, for the phenol-indole dimer) used to construct the radial scan. Each k unit corresponds to a 0.1 Å step along the intermolecular axis (defined in the “Generation of QM-based dimer geometries” section of the manuscript). These steps are, in general, taken in both the negative (more compact) and positive (more separated) directions with respect to the reference geometry (k = 0). All circles shown on the CCSD(T) curve correspond to data points included in the DES370K dataset. Red circles on the CCSD(T) curve correspond to data points included in the DES15K dataset. In the case of QM-derived dimer scans (as is the case here), we selected four conformers to include in DES15K: the conformer with the lowest energy, designated *E*_min_; the conformer that was less compact than the lowest-energy conformer and had an energy nearest to *E*_min_ + 0.5*E*_exc_, where the positive excitation energy is defined as *E*_exc_ = min(|*E*_min_|, 10 kcal mol^−1^); the conformer representing the zero of the interaction energy curve (when |*E*_min_| < 10 kcal mol^−1^), such that it was more compact than the lowest-energy conformer with an energy nearest to *E*_min_ + *E*_exc_; and the conformer with an energy nearest to *E*_min_ + 3*E*_exc_, which is representative of the repulsive region of the radial scan. Figure made with Grapher™ (Golden Software, LLC; http://www.goldensoftware.com).

DES15K is a core subset of the most representative structures from DES370K, and was assembled with a focus on retaining the chemical and orientational diversity of DES370K. DES15K consists of dimer configurations from both QM- and MD-derived scans, though with reduced resolution of scan points in the radial profiles. In the case of QM-optimized dimers, up to four conformers were selected, all from the radial scan corresponding to the most stable QM-optimized structure. These conformers correspond to the minimum, a point less compact than the minimum, the zero-crossing point at a more compact structure (if the minimum is not too deep), and a point representative of the repulsive wall at short distances. The exact definition of these points is specified in Fig. 3, which shows a typical dimer interaction energy profile, highlighting points along the scan that are included in DES15K. The DES15K dimers extracted from MD simulations include up to 10 conformers, and in addition to the MD-observed dimer, the minima along the corresponding radial scans at least half as deep as the most stable QM-optimized configuration. Based on these selection criteria, the MD-based component of DES15K includes only dimers for which we have the corresponding QM-optimized structure and sufficiently attractive scans. DES15K thus features a smaller set of monomers than DES370K, though most of the removed monomers are alkylated forms of the monomers still included in DES15K (and so the chemical diversity of the dimers—that is, at the level of functional-group interactions—is largely maintained).

## Data Records

Datasets are provided as CSV files (one file each for DES370K, DES15K, DES5M, DESS66, and DESS66x8) in a Figshare data repository^42^. A table providing column names and a description of the contents of each column can be found in the Supplementary Information. The Pandas^87^ and Scipy and Numpy^88^ packages were used in data processing and packaging for the CSV files.

## Technical Validation

### Validation of monomer geometries

Employing an established strategy^89^, we used molecular graph connectivity to confirm that the final set of dimer conformers had not undergone extreme or unintended changes in geometry during any stage of the protocols used to generate those geometries. Connectivity for each dimer, based on the original SMILES string, was compared against the graph assigned by Open Babel^45^ from the final atom positions of each conformer. Bond order, formal charge, and stereochemistry were ignored in order to avoid ambiguities in molecular graph construction. That is, we verified that the molecular graphs are isomorphic (i.e., they have identical edges between nodes labeled by element). We also calculated monomer energies with the OPLS_2005 force field^46^ and rejected dimers that included any monomer with excitation energy >30 kcal mol^−1^.

### Comparison to the S66 and S66x8 datasets

We applied the present protocol for estimating CCSD(T)/CBS dimer interaction energies to all 66 conformers from the S66 dataset^9^ and to all 528 conformers from the S66x8 dataset^8^, using the reference geometries in both cases. The results of these calculations are provided in the DESS66 and DESS66x8 datasets, respectively. We found good correspondence between our estimates and the values reported in Tables 6 and 7 of Kesharwani *et al*.^90^: mean unsigned errors of 0.07 kcal mol^−1^ (S66) and 0.05 kcal mol^−1^ (S66x8) and mean signed errors of −0.02 kcal mol^−1^ (S66) and −0.01 kcal mol^−1^ (S66x8).

### Comparison of bond-length constraints to published experimental data

To validate the present protocol for determining the values for bond constraints involving hydrogen atoms, we compared the values to published experimental data^91^. For the methyl (CH_3_) and methylene (CH_2_) functional groups, our values for the CH bond length—1.101 Å and 1.107 Å, respectively—compare favorably with the experimental distribution, which features a median value of 1.107 Å. We observed similarly good agreement between computed (1.098 Å) and experimental (median of 1.094 Å) bond lengths between hydrogen and aromatic carbon. The present approach yields values for NH bond lengths in primary and secondary amines of 1.026 Å and 1.028 Å, respectively, which are close to the experimentally measured length of 1.021 (±0.006) Å^91^. For bonds between hydrogen and oxygen, experimental uncertainties are even larger. For alcohols, our protocol yielded a bond length of 0.976 Å before the condensed-phase correction and 0.980 Å after the correction; the former value compares very well with the experimentally determined bond length of 0.975 (±0.010) Å in methanol^91^. In carboxylic acids, our approach yields a bond length of 0.983 Å before the condensed-phase correction and 0.996 Å after the correction; the former is comparable to the experimentally measured value of 0.981 (±0.003) Å for formic acid.

### Ionic system tests

The DES370K and DES5M collections include two types of ionic systems: dimers with only one of the monomers carrying a charge (−1, +1, or +2) and the other monomer neutral, and salts composed of a monovalent cation (+1) and a monovalent anion (−1). At large separations and in the absence of solvent, the desired biologically relevant monomer charges frequently do not represent the ground state. As a precautionary step, we clipped radial scans containing salts or divalent cations to separations between −0.5 and 0.5 Å from the reference structure used to seed the scan. We performed a natural population analysis (NPA)^92^, implemented in Molpro 2015.1 (http://www.molpro.net)^59^, to confirm that the interaction energies corresponded to the desired charges. We required that the NPA charges of each monomer differed from the target by <0.3 electrons, were smooth (as described in the next section), and approached the correct asymptotic limit.

### Smoothness and curvature tests for radial scans

For each radial scan, we imposed two additional conditions, requiring both that the interaction energy and all components were smooth functions of the separation and that they asymptotically converged to zero at large distances. The smoothness was validated by fitting each radial profile with a cubic spline and assessing the impact of individually removing each data point from the fit. In addition, we ensured that along a given scan, the total interaction energy featured no more than one local minimum. Scans without a local minimum were considered valid only if the interaction energy was strictly positive. Scans with a negative local minimum were allowed to exhibit at most one local maximum with a positive interaction energy.

## Supplementary information

Supplementary Information
