# Supplementary material for: Quantum chemical benchmark databases of gold-standard dimer interaction energies
Source: Sci Data. 2021 Feb 10;8:55. doi: 10.1038/s41597-021-00833-x (PMC7876112; doi:10.1038/s41597-021-00833-x)
Supplement: Supplementary file 1 — Supplementary Information [file 41597_2021_833_MOESM1_ESM.pdf]

# Supplementary Information
# Quantum chemical benchmark databases of gold-standard dimer interaction energies

## Basis sets

To provide as consistent a description of our systems as possible, we assembled a set of orbital and auxiliary basis sets from the literature that represented the best combination we could find for each element. We performed correlated calculations and wanted to be able to systematically extrapolate the results, and so we focused on Dunning-type, correlation-consistent orbital basis sets. Results are summarized in Table S1, though we discuss details below.

### *Orbital basis sets*

For hydrogen, helium, and the second row p-block elements (C, N, O, F, Ne) we used the standard (for intermolecular interactions) aug-cc-pVXZ[1–3] orbital basis sets. For third row p-block elements (P, S, Cl, Ar), we used the revised aug-cc-pV(X+d)Z[4,5] basis sets, which include an additional tight d-function and converge more rapidly to the complete-basis-set limit.

For the second and third row s-block elements (Li, Na, Mg, K, Ca), we considered only interactions where these elements are in their cationic form, corresponding to their primary biological state. That means that the neutral valence shell is empty and that the outer-core orbitals are the relevant electronic degrees of freedom. To ensure that we properly correlated

those electrons, we used the reweighted core-valence basis sets (cc-pwCVXZ),[6–8] without additional diffuse functions, which would be unnecessary for cations.

Beyond the third row, we recognized the importance of relativistic effects for the inner-core electrons, and rather than treating those effects explicitly, we replaced them with effective core potentials. We used the spin-orbit Dirac-Fock inner-core effective core potentials (ECPs)[9–10] (ECPnMDF, where *n* is the number of replaced electrons). For the outer-core and valence electrons, we used the corresponding correlation-consistent basis sets designed to work with pseudopotentials, (aug-cc-pwCVXZ-PP[11–13]) as basis sets. For SAPT0 calculations, the version of Psi4 that was available did not support ECPs. For these calculations, we took the perspective that the interaction would be dominated by the valence and that we could use, with minor qualms, the all-electron aug-cc-pwCVXZ[14,15] basis set.

In the main text, we denote all of these variations as "aVXZ" even if the basis set for a given element does not include augmented functions.

### *Auxiliary basis sets*

Whenever possible, we used the Hartree-Fock and MP2 auxiliary basis sets that were developed to match the orbital basis sets discussed above;[16] in several cases, however, these were not available. In particular, for He, Li, Na, Mg, K, Ca, Br, Kr, I, and Xe, no Hartree-Fock (JK) auxiliary basis was available for the correlation-consistent basis sets. Instead, we used the auxiliary basis sets for the largest of Turbomole def2 family of basis sets (def2-QZVPP), augmented when appropriate (def2-AQZVPP[17]). These were developed to be universal and apply not just to the def2 orbital basis sets but also the correlation-consistent basis sets. For

potassium and calcium, we needed to identify an MP2 auxiliary basis as well. Since none was available in the literature, we used the conservative approach of uncontracting the cc-pwCVXZ basis for X one cardinal number higher than the orbital basis.

***Definition of frozen core orbitals***

For correlated calculations, such as MP2, CCSD(T), and SAPT0, the choice of which core (spatial) orbitals to freeze is a complex problem. When calculating interaction energies, this is even more challenging, because of the possibility of inconsistent behavior between the monomer and dimer calculations. In addition, our calculations involved atoms in various charge states as well as the neutral state, so which electrons were "valence" and which were "core" did not always agree with the assumptions made by most quantum chemistry packages.

To automate the choice, we ran a series of large-basis set Hartree-Fock calculations on atoms, and used the resultant orbital energies to define cutoffs that both matched chemical intuition and provided sufficiently large energy gaps to make unambiguous decisions that would not rely on details of the calculation (either the exact interaction that we were calculating or the differences in basis set or correlation method). These investigations led to the following assignments (summarized in Table S1): 0 frozen orbitals for elements H–Li; 1 frozen orbital for elements C–Mg; 5 frozen orbitals for elements P–Ca; 4 frozen orbitals for Br, Kr, I, and Xe, where an ECP is present (i.e. in MP2 or CCSD(T) calculation); 9 frozen orbitals for Br and Kr without an ECP; and 18 frozen orbitals for I and Xe without an ECP. These choices differ slightly from the defaults in most quantum packages, particularly in the handling of Li, Na, Mg, K, and Ca, for which we did not freeze the outer-core electrons (the valence when the atom is in its charged state.

| Element | Orbital basis | Hartree-Fock auxiliary basis | MP2 auxiliary basis | Number of frozen core orbitals |
|---|---|---|---|---|
| H | aug-cc-pV*X*Z | aug-cc-pV*X*Z | aug-cc-pV*X*Z | 0 |
| He | aug-cc-pV*X*Z | def2-AQZVPP | aug-cc-pV*X*Z | 0 |
| Li | cc-pwCV*X*Z | def2-QZVPP | cc-pwCV*X*Z | 0 |
| C, N, O, F, Ne | aug-cc-pV*X*Z | aug-cc-pV*X*Z | aug-cc-pV*X*Z | 1 |
| Na, Mg | cc-pwCV*X*Z | def2-QZVPP | cc-pwCV*X*Z | 1 |
| P, S, Cl, Ar | aug-cc-pV(*X*+*d*)Z | aug-cc-pV*X*Z | aug-cc-pV*X*Z | 5 |
| K, Ca | cc-pwCV*X*Z | def2-QZVPP | Uncontracted cc-pwCV(*X*+1)Z | 5 |
| Br, Kr (MP2/CCSD(T)) | aug-cc-pwCV*X*Z-PP + ECP10MDF | def2-AQZVPP | aug-cc-pwCV*X*Z-PP | 4 |
| Br, Kr (SAPT0) | aug-cc-pwCV*X*Z | def2-AQZVPP | aug-cc-pwCV*X*Z | 9 |
| I, Xe (MP2/CCSD(T)) | aug-cc-pwCV*X*Z-PP + ECP28MDF | def2-AQZVPP | aug-cc-pwCV*X*Z-PP | 4 |
| I, Xe (SAPT0) | aug-cc-pwCV*X*Z | def2-AQZVPP | aug-cc-pwCV*X*Z | 18 |

**Table S1.** Orbital basis set, auxiliary basis sets, and number of frozen core spatial orbitals for all elements present in the dataset.

## Molecule classes and associated SMILES strings for DES370K

acids (count = 4)

CCC(=O)O, CC(=O)O, OC=O, OC(=O)CC(=O)O

alcohols (count = 10)

CCCO, CCC(O)C, CCO, CC(O)C, CO, OC1CCCC1, OC1CCCCC1, OCCCCO, OCCCO, OCCO

alkanes (count = 14)

C1CCCC1, C1CCCCC1, C, CC1CCCC1, CC1CCCCC1, CC, CCC, CC(C)C, CCCC, CC(C)(C)C, CCC(C)C, CCCCC, CCC(C)(C)C, CCCCCC

alkenes (count = 12)

C=C, CC=C, CC=CC, CC(=C)C, CCC=C, CC=C(C)C, CCC=CC, CCC(=C)C, CC(=C(C)C)C, CCC=C(C)C, CCC(=CC)C, CCC(=C(C)C)C

amides (count = 28)

CCCNC=O, CCC(=O)N, CCC(=O)NC, CCC(=O)N(C)C, CCC(=O)N(CC)C, CCNC=O, CCNC(=O)C, CCN(C=O)CC, CCN(C(=O)C)C, CCNC(=O)CC, CCN(C(=O)C)CC, CC(=O)N, CC(=O)N(C)C, CNC=O, CNC(=O)C, CN(C=O)CC, CNC(=O)CC(=O)N, CNC(=O)CC(=O)NC, CNC(=O)CNC=O, CNC(=O)CNC(=O)C, CNC(=O)C(NC(=O)C)C, NC=O, NC(=O)CC(=O)N, O=CN(C)C, O=CNCCC(=O)N, O=CNCCC(=O)NC, O=CNCCNC=O, O=CNCC(=O)N

amines (count = 20)

C1CCCN1, C1CCCNC1, CCCN, CCCNC, CCCN(C)C, CCN, CCN(C)C, CCNCC, CCN(CC)C, CN, CNC, CN(C)C, CNCC, CNCCCN, CNCCCNC, CNCCN, CNCCNC, N, NCCCN, NCCN

ammoniums (count = 6)

CC[NH3+], C[N+](C)(C)C, C[NH2+]C, C[NH3+], C[NH+](C)C, [NH4+]

benzene (count = 3)

c1ccccc1, Cc1ccccc1, CCc1ccccc1

carboxylates (count = 3)

[O-]C=O, [O-]C(=O)C, [O-]C(=O)CC

esters (count = 10)

CCCOC=O, CCC(=O)OC, CCOC(=O)CC, CCOC(=O)C, CCOC=O, COC(=O)C, COC=O, O=COCCCOC=O, O=COCCOC=O, O=COCOC=O

ethers (count = 15)

C1CCCO1, C1CCCOC1, C1CCOCO1, C1OCCO1, CCCOC, CCCOCOC, CCOCC, COCCCOC, COCC, COCCOC, COC, COCOCC, COCOC, O1CCOCC1, O1COCOC1

guanidiniums (count = 3)

CCNC(=[NH2+])N, CNC(=[NH2+])N, NC(=[NH2+])N

imidazolium (count = 3)

c1[nH]cc[nH+]1, Cc1c[nH]c[nH+]1, CCc1c[nH]c[nH+]1

ketones (count = 6)

CCC(=O)CC, CCC(=O)C, CCC=O, CC(=O)C, CC=O, C=O

monoatomics (count = 14)

[Ar], [Br-], [Ca+2], [Cl-], [F-], [He], [I-], [K+], [Kr], [Li+], [Mg+2], [Na+], [Ne], [Xe]

other (count = 191)

Brc1ccc(cc1)Br, Brc1ccccc1, BrC(Br)Br, BrCBr, BrCCBr, CBr, CC(Br)Br, CCBr, CCCC#CC, CCCC(Cl)(Cl)Cl, CCCC(Cl)Cl, CCCCCl, CCC#CC, CCCC#C, CCCC(F)(F)F, CCCC(F)F, CCCCF, CCC(Cl)(Cl)Cl, CCC(Cl)Cl, CCCCl, CCCC#N, CC#CC, CCC#C, CCC(F)(F)F, CCC(F)F, CCCF, CC(Cl)(Cl)Cl, CC(Cl)Cl, CCCl, CCC#N, CC#C, CC(F)(F)F, CC(F)F, CCF, CC(I)I, CCI, CCl, CC#N, CCOP(=O)(OC)OC, CCOP(=O)(OC)[O-], CCOP(=O)(OC)O, C#C, CF, CI, Clc1ccc(cc1)Cl, Clc1cccc(c1)Cl, Clc1ccccc1Cl, Clc1ccccc1, Clc1cc(Cl)c(c(c1Cl)Cl)Cl, Clc1cc(Cl)cc(c1)Cl, Clc1c(Cl)c(Cl)c(c(c1Cl)Cl)Cl, ClC(C(Cl)(Cl)Cl)(Cl)Cl, ClC(C(Cl)(Cl)Cl)Cl, ClCC(Cl)(Cl)Cl, ClCC(Cl)Cl, ClCCCl, ClC(Cl)Cl, ClCCl, CNCCCOC=O, CNCCCOC, CNCCC(=O)NC, CNCCC(=O)N, CNCCC(=O)O, CNCCCO, CNCCCSC, CNCCCS, CNCCNC=O, CNCCOC=O, CNCCOC, CNCC(=O)NC, CNCC(=O)N, CNCC(=O)O, CNCCO, CNCCSC, CNCCS, CNC(=O)CCN, CNC(=O)CC(=O)O, CNC(=O)CCO, CNC(=O)CCS, CNC(=O)CN, CNC(=O)COC=O, CNC(=O)CO, CNCOC=O, CNCOC, CNC(=O)CS, CNCSC, C#N, COCCCN, COCCCOC=O, COCCC(=O)NC, COCCC(=O)N, COCCC(=O)O, COCCCO, COCCCSC, COCCCS, COCCNC=O, COCCN, COCCOC=O, COCC(=O)NC, COCC(=O)N, COCC(=O)O, COCCO, COCCSC, COCCS, COCNC=O, COCN, COCOC=O, COCO, COCSC, COCS, COP(=O)(OC)OC, COP(=O)(OC)[O-], COP(=O)(OC)O, COP(=O)(O)O, COP(=O)(OP(=O)(O)O)[O-], CSCCCNC=O, CSCCCN, CSCCCOC=O, CSCCC(=O)N, CSCCC(=O)O, CSCCCO, CSCCN, CSCCOC=O, CSCC(=O)NC, CSCC(=O)N, CSCC(=O)O, CSCCO, CSCNC=O, CSCN, CSCOC=O, CSCO, Fc1ccc(cc1)F, Fc1cccc(c1)F,

Fc1ccccc1F, Fc1ccccc1, Fc1cc(F)c(c(c1F)F)F, Fc1cc(F)cc(c1)F, Fc1c(F)c(F)c(c(c1F)F)F, FC(C(F)(F)F)(F)F, FC(C(F)(F)F)F, FCC(F)(F)F, FCC(F)F, FCCF, FC(F)F, FCF, ICCI, ICI, NCCCOC=O, NCCC(=O)N, NCCC(=O)O, NCCCO, NCCCS, NCCNC=O, NCCOC=O, NCC(=O)N, NCC(=O)O, NCCO, NCCS, NC(=O)CC(=O)O, NC(=O)CCO, NC(=O)CCS, NC(=O)CO, NCOC=O, NC(=O)CS, OCCCNC=O, OCCCOC=O, OCCC(=O)O, OCCCS, OCCNC=O, OCCOC=O, OCC(=O)O, OCCS, O=CNCCC(=O)O, O=CNCCOC=O, O=CNCC(=O)O, O=CNCOC=O, O=COCCC(=O)NC, O=COCCC(=O)N, O=COCCC(=O)O, O=COCC(=O)N, O=COCC(=O)O, OC(=O)CCS, OCOC=O, OC(=O)CS, OP(=O)(O)O, [O-]P(=O)(OP(=O)(OC)O)O, SCCCOC=O, SCCNC=O, SCCOC=O, SCOC=O, [H][H]

phenol (count = 3)

Cc1ccc(cc1)O, CCc1ccc(cc1)O, Oc1ccccc1

pyridine (count = 3)

c1cccnc1, c1ccncn1, n1ccncc1

pyrrole (count = 9)

c1ccc2c(c1)[nH]cc2, c1ccc[nH]1, c1ncc[nH]1, Cc1cnc[nH]1, Cc1c[nH]c2c1cccc2, Cc1c[nH]cn1, CCc1cnc[nH]1, CCc1c[nH]c2c1cccc2, CCc1c[nH]cn1

sulfides (count = 21)

C1CCCS1, C1CCCSC1, C1CCSCS1, C1CCSSC1, C1CSSC1, C1SCCS1, CCCSCSC, CCCSC, CCCSSC, CCSCC, CCSSCC, CCSSC, CSCCCSC, CSCCSC, CSCC, CSCSCC, CSCSC, CSC, CSSC, S1CCSCC1, S1CSCSC1

thiols (count = 13)

CCCSS, CCCS, CCSS, CCS, CSCCCS, CSCCS, CSCS, CSS, CS, SCCCS, SCCS, SS, S

water (count = 1)

O

## Description of columns in database

| Column name | Column index | | Description of contents |
|---|---|---|---|
| | DES370K, DES15K, DESS66, & DESS66x8 | DES5M | |
| smiles0 | 0 | 0 | SMILES string of the first monomer |
| smiles1 | 1 | 1 | SMILES string of the second monomer |
| charge0 | 2 | 2 | Charge of the first monomer |
| charge1 | 3 | 3 | Charge of the second monomer |
| natoms0 | 4 | 4 | Number of atoms in the first monomer |
| natoms1 | 5 | 5 | Number of atoms in the second monomer |
| system_id | 6 | 6 | Identifier for each unique dimer system |
| group_orig | 7 | 7 | Generation method for a group of geometries: "qm_opt_dimer", "md_dimer", "md_nmer", "md_solvation" |
| group_id | 8 | 8 | Identifier for a group of geometries originating from a common reference geometry, either (1) points along a radial scan, or (2) dimers belonging to a cluster |
| k_index | 9 | 9 | Number of 0.1-Å offsets from a common reference geometry (from a radial scan, -50 <= k_index <= 40) or a unique identifier for a specific dimer geometry (from a cluster, k_index >= 100) |
| geom_id | 10 | 10 | Identifier for a single unique geometry |
| cc_basis | 11 | N/A | Basis set used in the CCSD(T) calculation |
| cc_HF | 12 | N/A | Hartree-fock interaction energy (from the CCSD(T) calculation) |
| cc_MP2_os | 13 | N/A | Singlet (opposite-spin) MP2 correlation interaction energy from the CCSD(T) calculation |
| cc_MP2_ss | 14 | N/A | Triplet (same-spin) MP2 correlation interaction energy from the CCSD(T) calculation |
| cc_CCSD_os | 15 | N/A | Singlet (opposite-spin) CCSD correlation interaction energy |

| | | | |
|---|---|---|---|
| cc_CCSD_ss | 16 | N/A | Triplet (same-spin) CCSD correlation interaction energy |
| cc_(T) | 17 | N/A | (T) contribution to the correlation interaction energy |
| cc_[T] | 18 | N/A | [T] contribution to the correlation interaction energy |
| cc_MP2_all | 19 | N/A | Total MP2 interaction energy from the CCSD(T) calculation, i.e., “cc_MP2_os + cc_MP2_ss + cc_HF” |
| cc_CCSD(T)_all | 20 | N/A | Total CCSD(T) interaction energy, i.e., “cc_CCSD_os + cc_CCSD_ss + cc_(T) + cc_HF” |
| cc_delta_CCSD(T) | 21 | N/A | Post-MP2 CCSD(T) correlation interaction correction, i.e., "cc_CCSD(T)_all - cc_MP2_all" |
| qz_HF | 22 | 11 | Hartree-Fock interaction energy |
| qz_MP2_os | 23 | 12 | Singlet (opposite-spin) MP2 correlation interaction energy |
| qz_MP2_ss | 24 | 13 | Triplet (same-spin) MP2 correlation interaction energy |
| qz_MP2_all | 25 | 14 | Total MP2 interaction energy, i.e., “qz_MP2_os + qz_MP2_ss + qz_HF” |
| tz_HF | 26 | 15 | Hartree-Fock interaction energy |
| tz_MP2_os | 27 | 16 | Singlet (opposite-spin) MP2 correlation interaction energy |
| tz_MP2_ss | 28 | 17 | Triplet (same-spin) MP2 correlation interaction energy |
| tz_MP2_all | 29 | 18 | Total MP2 interaction energy, i.e., “tz_MP2_os + tz_MP2_ss + tz_HF” |
| cbs_MP2_os | 30 | 19 | Singlet (opposite-spin) MP2 correlation interaction energy (CBS extrapolation from av[TQ]z) |
| cbs_MP2_ss | 31 | 20 | Triplet (same-spin) MP2 correlation interaction energy (CBS extrapolation from av[TQ]z) |
| cbs_MP2_all | 32 | 21 | Total CBS MP2 interaction energy, i.e., "qz_HF + cbs_MP2_os + cbs_MP2_ss" |
| cbs_CCSD(T)_all | 33 | N/A | Total CBS CCSD(T) interaction energy, i.e., "cbs_MP2_total + cc_delta_CCSD(T)" |
| espx_HF_es | 34 | 22 | Hartree-Fock electrostatic interaction energy |
| espx_HF_hl | 35 | 23 | Hartree-Fock Heitler-London interaction energy |
| espx_HF_ovl | 36 | 24 | Hartree-Fock density matrix overlap |
| espx_MP2_es | 37 | 25 | MP2 electrostatic interaction energy |
| espx_MP2_ovl | 38 | 26 | MP2 density matrix overlap |
| sapt_es | 39 | 27 | SAPT0 first-order electrostatic energy |
| sapt_ex | 40 | 28 | SAPT0 first-order exchange energy |

| | | | |
|---|---|---|---|
| sapt_exs2 | 41 | 29 | SAPT0 first-order exchange energy (S^2 approximation) |
| sapt_ind | 42 | 30 | SAPT0 second-order induction energy |
| sapt_exind | 43 | 31 | SAPT0 second-order exchange-induction energy |
| sapt_disp | 44 | 32 | SAPT0 second-order dispersion energy |
| sapt_exdisp_os | 45 | 33 | SAPT0 singlet (opposite-spin) second-order exchange-dispersion energy |
| sapt_exdisp_ss | 46 | 34 | SAPT0 triplet (same-spin) second-order exchange-dispersion energy |
| sapt_delta_HF | 47 | 35 | Difference between the HF interaction energy and the lowest-order SAPT0 interaction terms |
| sapt_all | 48 | 36 | Total SAPT0 interaction energy, i.e., "sapt_es + sapt_ind + sapt_exind + sapt_delta_HF + sapt_disp + sapt_exdisp_os + sapt_exdisp_ss" |
| nn_CCSD(T)_all | 49 | 37 | SNS-MP2 predicted total CBS CCSD(T) interaction energy |
| nn_CCSD(T)_all_05 | 50 | 38 | 5th percentile SNS-MP2 predicted total CBS CCSD(T) interaction energy |
| nn_CCSD(T)_all_95 | 51 | 39 | 95th percentile SNS-MP2 predicted total CBS CCSD(T) interaction energy |
| xyz | 52 | 40 | Atomic positions ordered as: x0, y0, z0, x1, y1, z1, ..., xn, yn, zn |
| elements | 53 | 41 | Atoms present in the system, in the same order as the coordinates in "xyz" |
| reference | 54 | N/A | Silver or bronze-standard reference interaction energy, (present in DESS66 and DESS66x8 only) |
| system_name | 55 | N/A | Canonical S66 or S66x8 system name (present in DESS66 and DESS66x8 only) |

**Table S2.** Columns starting with "cc_" refer to properties of the CCSD(T) calculation, and used a variable basis set identified by the column "cc_basis." Columns starting with "qz_" and "espx_" refer to properties of MP2/aVQZ calculations, while columns starting with "tz_" refer to properties of aVTZ calculations. Columns starting with "sapt_" refer to properties of the SAPT0 calculation using the aVTZ basis set. Columns starting with "cbs_" refer to properties of a aV[TQ]Z complete basis set (CBS) approximation (using a two-point Helgaker extrapolation of the MP2 correlation energy). Columns starting with "nn_" refer to properties of SNS-MP2

predicted energies, which are fitted to the CBS approximation. Columns ending with "_all" refer to a complete interaction energy containing both the Hartree-Fock (HF) interaction energy and any applicable correlation energies and corrections.

## Supplemental References


1. Dunning Jr., T.H. Gaussian basis sets for use in correlated molecular calculations. I. The atoms boron through neon and hydrogen. *J. Chem. Phys.* **90**, 1007–1023 (1989).

2. Woon, D.E. & Dunning Jr., T.H. Gaussian basis sets for use in correlated molecular calculations. IV. Calculation of static electrical response properties. *J. Chem. Phys.* **100**, 2975–2988 (1994).

3. Kendall, R.A., Dunning Jr., T.H. & Harrison, R.J. Electron affinities of the first-row atoms revisited. Systematic basis sets and wave functions. *J. Chem. Phys.* **96**, 6796–6806 (1992).

4. Woon, D.E. & Dunning Jr., T.H. Gaussian basis sets for use in correlated molecular calculations. III. The atoms aluminum through hydrogen. *J. Chem. Phys.* **98**, 1358–1371 (1993).

5. Dunning Jr., T.H., Peterson, K.A. & Wilson, A.K. Gaussian basis sets for use in correlated molecular calculations: X. The atoms aluminum through argon revisited. *J. Chem. Phys.* **114**, 9244–9253 (2001).

6. Peterson, K.A. & Dunning Jr., T.H. Accurate correlation consistent basis sets for molecular core–valence correlation effects: The second row atoms Al–Ar, and the first row atoms B–Ne revisited. *J. Chem. Phys.* **117**, 10548–10560 (2002).

7. Prascher, B., Woon, D.E., Peterson, K.A., Dunning Jr., T.H. & Wilson, A.K. Gaussian basis sets for use in correlated molecular calculations. VII. Valence, core-valence, and scalar relativistic basis sets for Li, Be, Na, and Mg. *Theor. Chem. Acc.* **128**, 69–82 (2011).

8. Koput, J. & Peterson, K.A. Ab initio potential energy surface and vibrational-rotational energy levels of $X^2\Sigma^+$ CaOH. *J. Phys. Chem. A* **106**, 9595–9599 (2002).

9. Lim, I.S., Schwerdtfeger, P., Metz, B. & Stoll, H. All-electron and relativistic pseudopotential studies for the group 1 element polarizabilities from K to element 119. *J. Chem. Phys.* **122,** 104103 (2005).

10. Lim, I.S., Stoll, H. & Schwerdtfeger, P. Relativistic small-core energy-consistent pseudopotentials for the alkaline-earth elements from Ca to Ra. *J. Chem. Phys.* **124**, 034107 (2006).

11. Peterson, K.A. & Yousaf, K.E. Molecular core-valence correlation effects involving the post-d elements Ga-Rn: benchmarks and new pseudopotential-based correlation consistent basis sets. *J. Chem. Phys.* **133**, 174116 (2010).

12. Peterson, K.A., Shepler, B.C., Figgen, D. & Stoll, H. On the spectroscopic and thermochemical properties of ClO, BrO, IO, and their anions. *J. Phys. Chem. A* **110**, 13877–13883 (2006).

13. Peterson, K.A., Figgen, D., Goll, E., Stoll, H. & Dolg, M. Systematically convergent basis sets with relativistic pseudopotentials. II. Small-core pseudopotentials and correlation consistent basis sets for the post-d group 16–18 elements. *J. Chem. Phys.* **119**, 11113–11123 (2003).

14. Wilson, A.K., Woon, D.E., Peterson, K.A. & Dunning Jr., T.H. Gaussian basis sets for use in correlated molecular calculations. IX. The atoms gallium through krypton. *J. Chem. Phys.* **110**, 7667–7676 (1999).

15. DeYonker, N.J., Peterson, K.A. & Wilson, A.K. Systematically convergent correlation consistent basis sets for molecular core–valence correlation effects: the third-row atoms gallium through Krypton. *J. Phys. Chem. A* **111**, 11383–11393 (2007).

16. Weigend, F. A fully direct RI-HF algorithm: Implementation, optimized auxiliary basis sets, demonstration of accuracy and efficiency. *Phys. Chem. Chem. Phys.* **4**, 4285–4291 (2002).

17. Weigend, F. Hartree–Fock exchange fitting basis sets for H to Rn. *J. Comput. Chem.* **29**, 167–175 (2008).

18. Kesharwani, M.K., Karton, A., Sylvetsky, N. & Nitai, J.M.L. The S66 non-covalent interactions benchmark reconsidered using explicitly correlated methods near the basis set limit. *Aust. J. Chem.* **71**, 238–248 (2018).
